# Influence of Carbon Source on the Buffer Layer for 4H-SiC Homoepitaxial Growth

**DOI:** 10.3390/ma17112612

**Published:** 2024-05-29

**Authors:** Shangyu Yang, Ning Guo, Siqi Zhao, Yunkai Li, Moyu Wei, Yang Zhang, Xingfang Liu

**Affiliations:** 1 Key Laboratory of Semiconductor Materials Science, Institute of Semiconductors, Chinese Academy of Sciences, Beijing 100083, China; yangsy@semi.ac.cn (S.Y.); guoning@semi.ac.cn (N.G.); zhaosiqi@semi.ac.cn (S.Z.); liyunkai@semi.ac.cn (Y.L.); weimoyu@semi.ac.cn (M.W.); 2 College of Materials Science and Opto-Electronic Technology, University of Chinese Academy of Sciences, Beijing 100049, China; 3 Beijing Key Laboratory of Low Dimensional Semiconductor Materials and Devices, Beijing 100083, China

**Keywords:** 4H-SiC, CVD, buffer

## Abstract

In this study, we systematically explore the impact of C/Si ratio, pre-carbonization time, H_2_ etching time, and growth pressure on the buffer layer and subsequent epitaxial layer of 6-inch 4H-SiC wafers. Our findings indicate that the buffer layer’s C/Si ratio and growth pressure significantly influence the overall quality of the epitaxial wafer. Specifically, an optimal C/Si ratio of 0.5 and a growth pressure of 70 Torr yield higher-quality epitaxial layers. Additionally, the pre-carbonization time and H_2_ etching time primarily affect the uniformity and surface quality of the epitaxial wafer, with a pre-carbonization time of 3 s and an H_2_ etching time of 3 min found to enhance the surface quality of the epitaxial layer.

## 1. Introduction

Silicon carbide (SiC) materials have higher bandwidth, critical breakdown field strength, electron saturation drift rate, and higher thermal conductivity than traditional Si materials. In turn, the corresponding SiC devices have higher voltage resistance, high temperature resistance, and higher frequency, with high efficiency and energy-saving advantages, which have great potential for future applications and are highly expected in the green energy revolution [1,2,3,4,5,6]. The horizontal hot wall design of the susceptor is now the dominant design in CVD applications for SiC growth, as it provides more uniform heat distribution and better precursor cracking efficiency. Typically, SiC homoepitaxial growth is performed by chemical vapor deposition (CVD) using silane (SiH_4_) or TCS (SiHCl_3_) as silicon precursors and ethylene (C_2_H_4_) or propane (C_3_H_8_) as carbon precursors. Currently, SiC CVD epitaxial growth is grown by the step-controlled growth mode, which is favorable for obtaining the epitaxial layer with a single crystal type and reducing the growth temperature [7,8,9,10,11]. However, during the early stages of growth, scratches and contamination on the substrate surface can disrupt step-controlled growth, leading to the formation of 3C-SiC crystal types. Particularly in rapid epitaxial growth processes, excessive saturation of the growth source on the surface can promote two-dimensional nucleation, further resulting in the appearance of 3C-SiC crystals. Additionally, a significant doping concentration gradient between the substrate and the epitaxial layer may compromise interface quality. Introducing a buffer layer can effectively mitigate this gradient and enhance interface integrity. Previous studies have primarily focused on the growth process’s effects on the epitaxial layer, with limited research examining its impact on both the buffer and subsequent epitaxial layers [12,13,14,15,16,17,18,19,20,21]. This study systematically investigates the effects of the growth process on the buffer layer and subsequent epitaxial layer in the production of 6-inch 4H-SiC epitaxial wafers. The findings offer valuable insights for future research and production of 4H-SiC epitaxial layers.

## 2. Materials and Methods

A horizontal hot-wall chemical vapor deposition (CVD) system was utilized to explore the effects of the C/Si ratio, pre-carbonization time, H_2_ (Beijing He-Pu Beifen Gas Industry Co., Beijing, China) etching time, and growth pressure on the buffer and epitaxial layers of 6-inch 4H-SiC wafers. Ethylene (C_2_H_4_) and trichlorosilane (SiHCl_3_) served as carbon and silicon sources (Tianjin Lulin Gas Co., Tianjin, China), respectively. Firstly, the buffer layer process was grown to study the effect of C/Si ratio, the epitaxial conditions were growth temperature of 1570 °C, growth pressure of 70 Torr, C/Si ratio of 0.5, 0.53 and 0.56, respectively; and the effect of pre-carbonation time was studied, the epitaxial conditions were growth temperature of 1570 °C, growth pressure of 70 Torr, C/Si ratio of 0.53, and the pre-carbonation time of 2 s, 3 s, and 4 s. Then, the epitaxial layer process with buffer layer is studied to investigate the effect of buffer layer C/Si ratio on the epitaxial layer, and the epitaxial conditions are a growth temperature of 1570 °C, a pre-carbonization time of 3 s, and two growth pressures of 40 Torr and 70 Torr. The epitaxial layer C/Si ratio is fixed at 0.72, and the buffer layer C/Si ratios are 0.48, 0.53, 0.59, and 0.64; the H_2_ etching is studied to investigate the effect of H_2_ etching on the epitaxial layer, the growth pressure is 70 Torr, the C/Si ratio is 0.53, and the pre-carbonization time is 3 s. To study the effect of H_2_ etching time, the growth temperature is 1570 °C, the growth pressure is 70 Torr, the pre-carbonization time is 3 s, and the buffer layer and epitaxial layer C/Si ratio is 0.53 and 0.72, respectively. To study the effect of growth pressure, the growth temperature is 1570 °C, the pre-carbonization time is 3 s, the H_2_ etching time is 2 min, and the buffer layer and epitaxial layer C/Si ratio are 0.53 and 0.72, respectively, and the growth pressures were 60 Torr, 70 Torr, 75 Torr, and 80 Torr. The grown epitaxial wafers are of 4H-SiC single crystal, and the structural features with a similar structural motif in silicon carbide were previously confirmed [22].

Epilayer thickness was determined using Fourier transform infrared reflectance (FTIR, Nicolet IS50, Thermo Fisher, Waltham, MA, USA), with uniformity assessed by calculating the mean square deviation from thickness measurements at 33 uniformly distributed points on the wafer. Doping concentrations were analyzed through capacitance–voltage profiling (CV, MCV-530L Semilab, Budapest, Hungary), and doping uniformity was evaluated from 33 similarly distributed measurement points by examining variations in concentration data. The morphology of the epitaxial layers was analyzed using atomic force microscopy (AFM, AFM Dimension, Icon Bruker, Billerica, MA, USA) in tapping mode over a 10 μm × 10 μm in the center position of the substrate. Additionally, an Optical Surface Analyzer (Candela CS920, KLA Instruments, Milpitas, CA, USA) was employed to characterize surface morphology defects in the 4H-SiC epitaxial wafers.

## 3. Results

### 3.1. Effect of C/Si on the Buffer Layer

The effect of the buffer layer’s C/Si ratio on thickness uniformity, doping uniformity, and surface root mean square (rms) was examined. The growth conditions were controlled with a fixed temperature of 1570 °C and pressure of 70 Torr. The ethylene (C_2_H_4_) flow was maintained at 8 sccm, while the trichlorosilane (TCS) flow was adjusted for C/Si regulation. Nitrogen (N_2_) flow was set at 45 sccm, and the growth duration was 25 min.

**Figure 1:**
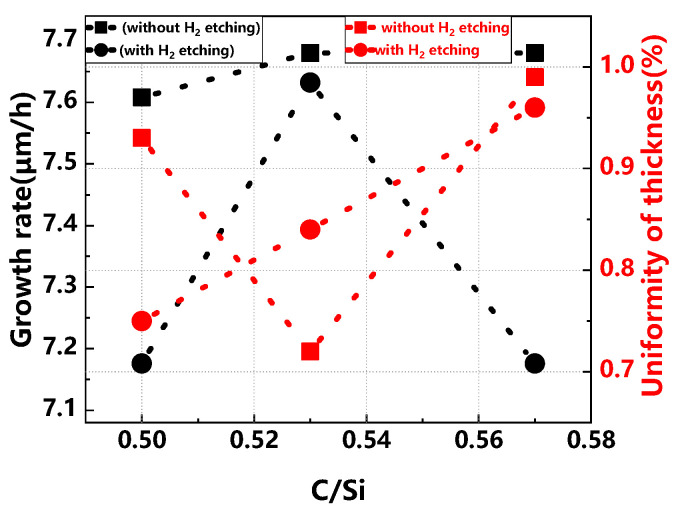
Comparison of buffer layer growth rate and thickness uniformity across various C/Si ratios, with and without H_2_ etching.

**Figure 2:**
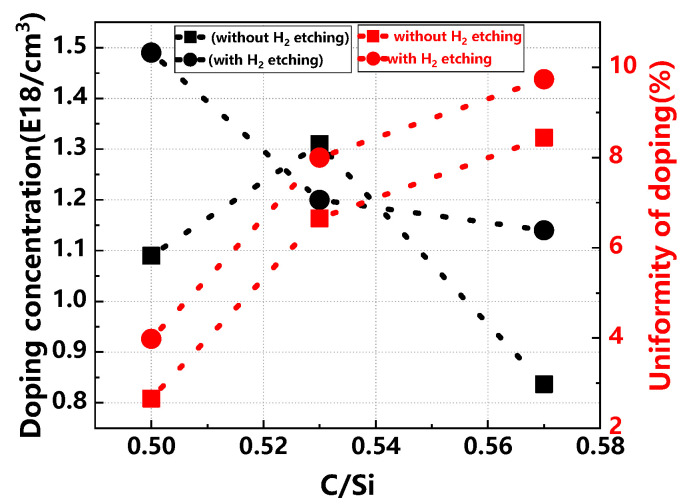
Comparison of buffer layer doping concentration and doping uniformity across various C/Si ratios, with and without H_2_ etching.

Figure 1 illustrates a comparison of the growth rate and thickness uniformity of buffer layers across various C/Si ratios, both with and without H_2_ etching. In the absence of H_2_ etching, the growth rate initially increases with the C/Si ratio before stabilizing due to a limitation imposed by the silicon source when the ratio exceeds 0.53. Conversely, with H_2_ etching, the growth rate first increases and then decreases as the C/Si ratio rises, exhibiting greater fluctuations and generally lower rates than those without etching. This decrease can be attributed to the effective removal of substrate defects by H_2_ etching, which also causes substrate depletion, impacting the buffer layer’s overall thickness and growth rate. Regarding thickness uniformity, it initially improves and then worsens without H_2_ etching as the C/Si ratio increases. With H_2_ etching, however, thickness uniformity shows a consistent linear decline, reflecting the etching’s influence in minimizing substrate surface quality variations, thus clarifying the relationship between thickness uniformity and the C/Si ratio [23,24]. Increasing the C/Si ratio facilitates the reaction between the carbon and silicon sources, thereby accelerating the epitaxial growth rate of SiC. However, excessively high C/Si ratios can induce surface morphology defects. Conversely, a lower C/Si ratio decelerates the growth rate due to a scarcity of the carbon source, and excessively low ratios may result in the formation of silicon droplets and pronounced surface steps. The C/Si ratio also has an important effect on the doping and doping uniformity of the buffer layer, and the specific trends are shown in Figure 2.

**Figure 3:**
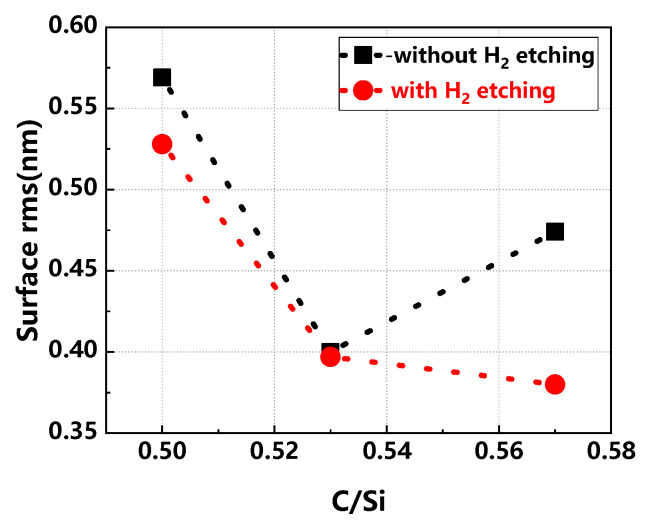
Comparison of surface rms in buffer layers across various C/Si ratios, with and without H_2_ etching.

Figure 3 demonstrates the comparison of surface rms value in buffer layers across various C/Si ratios, with and without H_2_ etching. In the absence of H_2_ etching, the rms value of the buffer layer surface decreases and then increases as the C/Si ratio increases. In the case of H_2_ etching, on the other hand, as the C/Si ratio increases, the rms value of the buffer layer surface continues to decrease, and the surface rms value is lower compared to the non-H_2_-etched condition. This indicates that the C/Si ratio has a significant effect on the growth and surface morphology of the buffer layer. At smaller C/Si ratios, it is beneficial to enhance the thickness uniformity of the epitaxial layer; meanwhile, smaller C/Si ratios also enhance the doping efficiency and doping uniformity of N_2_. However, at larger C/Si ratios, although the rms value of the buffer layer surface is reduced and a flatter surface is obtained, it may also adversely affect the doping uniformity. In addition, the introduction of H_2_ etching can effectively avoid the interference of the substrate surface quality on the experimental results and help to further reduce the rms value of the buffer layer surface [25].

### 3.2. Effect of Pre-Carbonization on the Buffer Layer

The surface polarity of the substrate critically affects the polymorphism of grown crystals. Given that the SiC lattice comprises a polar bilayer structure alternating between C and Si, it exhibits two distinct [0001] surfaces with differing surface energies: the [0001] Si surface and the [0001] C surface. The [0001] Si surface has higher surface energy, influencing crystal growth preferences. 4H-SiC which has a higher formation enthalpy, predominantly grows on the lower-energy C-surface, while 6H-SiC, with a lower formation enthalpy, favors the higher-energy Si-surface. To favor 4H-SiC formation and minimize 6H-SiC during epitaxial growth, we pre-carbonized the Si surface using a controlled C source strategy. However, the pre-carbonization duration is critical; excessive duration can lead to substrate over-carbonization, negatively impacting subsequent epitaxial growth. This study extensively investigates how pre-carbonization affects epitaxial wafer uniformity. Using a fixed growth temperature of 1570 °C, pressure of 70 Torr, C/Si ratio of 0.53, TCS flow rate of 30 sccm, N_2_ flow rate of 45 sccm, and growth duration of 25 min, we examined the impacts of pre-carbonization times of 2 s, 3 s, and 4 s on the uniformity of the single-buffer layer.

**Figure 4:**
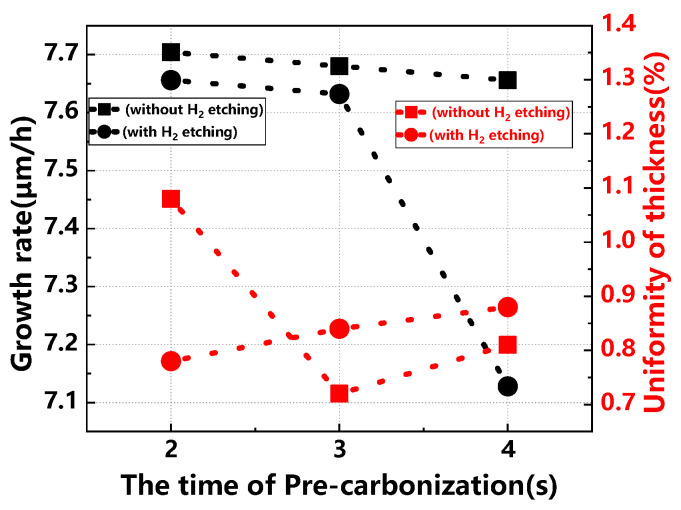
Buffer layer growth rate and thickness uniformity across different pre-carbonization times, with and without H_2_ etching.

Figure 4 details the effects of different pre-carbonization times on the buffer layer’s growth rate and thickness uniformity, with and without H_2_ etching. Without H_2_ etching, the growth rate slightly declines as pre-carbonization time increases, though the overall impact is minimal, suggesting a modest influence of pre-carbonization time on growth. Conversely, with H_2_ etching, the growth rate initially decreases slightly, then sharply declines with longer pre-carbonization times. Regarding thickness uniformity, without H_2_ etching, it initially improves but then worsens as the pre-carbonization time extends, reaching optimal uniformity at a 3 s pre-carbonization time. With H_2_ etching, thickness uniformity consistently worsens with increased pre-carbonization time, achieving the best uniformity at a 2 s pre-carbonization time.

**Figure 5:**
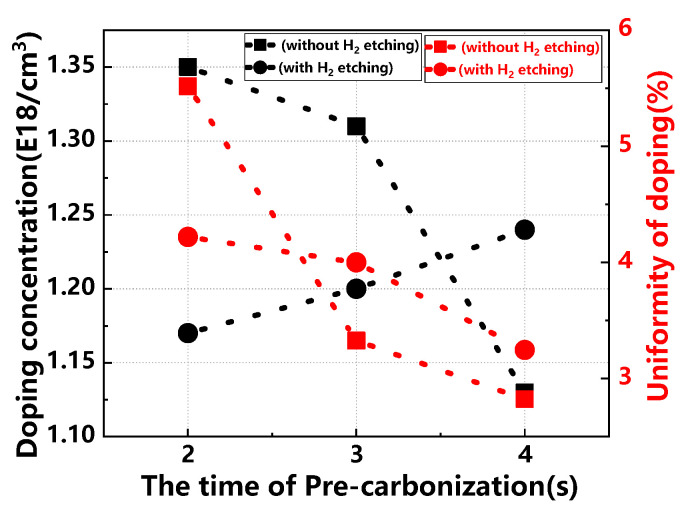
Buffer layer doping concentration and doping uniformity across different pre-carbonization times, with and without H_2_ etching.

Figure 5 illustrates how pre-carbonization times impact the doping concentration and uniformity in the buffer layer, with and without H_2_ etching. Without H_2_ etching, doping concentration decreases as pre-carbonization time increases. In contrast, with H_2_ etching, doping concentration consistently increases. Regarding doping uniformity, it improves with longer pre-carbonization times. Additionally, the introduction of H_2_ etching results in even greater uniformity in both doping concentration and uniformity across the samples.

**Figure 6:**
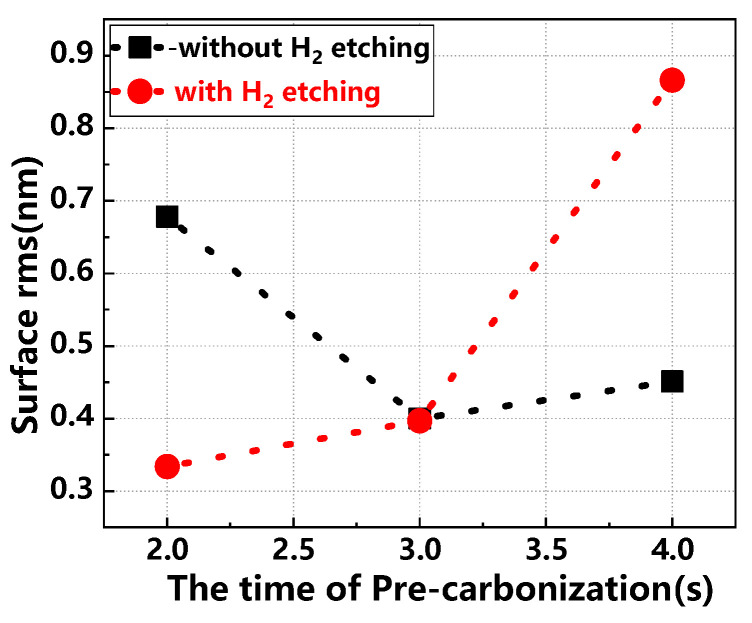
Surface rms values of the buffer layer for different pre-carbonization times, with and without H_2_ etching conditions.

Figure 6 details the surface rms values of the buffer layer under various pre-carbonization times, both with and without H_2_ etching. When H_2_ etching is applied, the rms value on the sample surface increases with longer pre-carbonization times, indicating a decline in surface quality. Without H_2_ etching, the rms value initially decreases, reaching its lowest at a pre-carbonization time of 3 s, which suggests optimal surface quality. Further analysis shows that pre-carbonization time minimally affects the growth rate and thickness uniformity, even with H_2_ etching. However, it significantly impacts doping concentration, doping uniformity, and surface rms value. Optimal buffer layer uniformity and surface quality are achieved at a pre-carbonization time of 3 s [26]. Optimal pre-carbonization duration significantly enhances the growth rate of subsequent epitaxial layers by reducing the nucleation barrier for SiC formation, thereby facilitating more rapid crystal growth. Insufficient pre-carbonization may lead to inadequate surface preparation, slowing nucleation and growth rates, while excessive pre-carbonization can create a thick carbon layer that obstructs active sites and disrupts growth interfaces, inhibiting SiC growth. Properly conducted pre-carbonization yields a uniform and reactive surface, promoting consistent growth across the wafer and resulting in epitaxial layers with uniform thickness. Conversely, inadequate pre-carbonization can produce an uneven surface, leading to variable layer thickness as growth preferentially occurs at adequately carbonized locations. The surface condition after carbonization also influences dopant incorporation during epitaxial growth. A smooth, uniformly carbonized surface ensures consistent dopant distribution, enhancing electrical uniformity and wafer performance. Furthermore, appropriate pre-carbonization minimizes surface defects like pits and scratches, which arise from mismatches in thermal expansion coefficients and lattice parameters. However, over-carbonization may increase surface roughness due to excessive carbon accumulation.

### 3.3. Effect of Buffer Layer C/Si on Epitaxial Layer

Growth experiments on epitaxial wafers with buffer layers aimed to assess the impact of the buffer layer’s C/Si ratio on the epitaxial layer. Based on earlier findings, specific growth conditions were established: a constant temperature of 1570 °C, pressure of 70 Torr, and pre-carbonization time of 3 s. We tested C/Si ratios of 0.48, 0.53, 0.59, and 0.64, maintaining the TCS flow rate at 30 sccm and the growth duration at 7 min. For the epitaxial layer, the temperature remained at 1570 °C, with trials conducted at two pressures, 40 Torr and 70 Torr. The experiments fixed the C/Si ratio at 0.72, with a TCS flow rate of 50 sccm and a growth time of 18 min.

**Figure 7:**
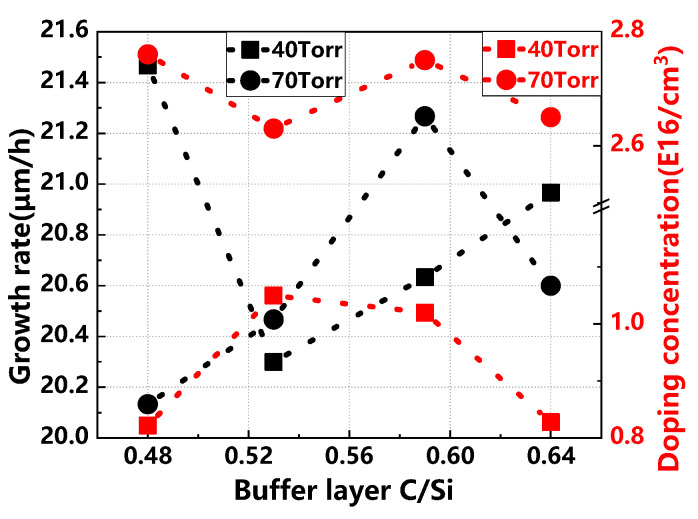
Growth rate and doping concentration of epitaxial layers at different buffer layer C/Si ratios and growth pressures.

Figure 7 illustrates the variations in growth rate and doping concentration of epitaxial layers under different buffer layer C/Si ratios and growth pressures. At 40 Torr, as the buffer layer C/Si ratio increases, the growth rate and doping concentration exhibit opposite trends: the growth rate initially decreases and then increases, whereas the doping concentration increases and then decreases. This pattern suggests that faster growth rates may hinder the dopant’s diffusion and doping process, resulting in lower doping concentrations. Notably, the growth rate of the buffer layer is generally lower than that of the epitaxial layer, which is beneficial for enhancing buffer layer quality and providing an optimal surface for epitaxial growth. At 70 Torr, the growth rate initially rises with increasing C/Si ratio before declining, while the doping concentration varies minimally.

**Figure 8:**
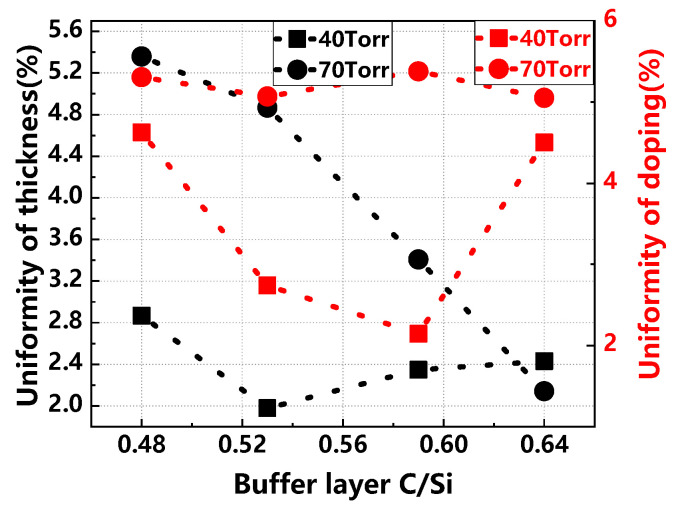
Thickness uniformity and doping uniformity of epitaxial layers with different buffer layer C/Si ratios and growth pressures.

Figure 8 depicts the impact of different buffer layer C/Si ratios and growth pressures on the thickness and doping uniformity of the epitaxial layer. At 40 Torr, the thickness uniformity of the epitaxial layer slightly fluctuates with changes in the buffer layer C/Si ratio. In contrast, at 70 Torr, the thickness uniformity shows a nearly linear improvement. Regarding doping uniformity, it remains stable at 70 Torr as the buffer layer C/Si ratio increases. However, at 40 Torr, doping uniformity improves initially but then deteriorates.

**Figure 9:**
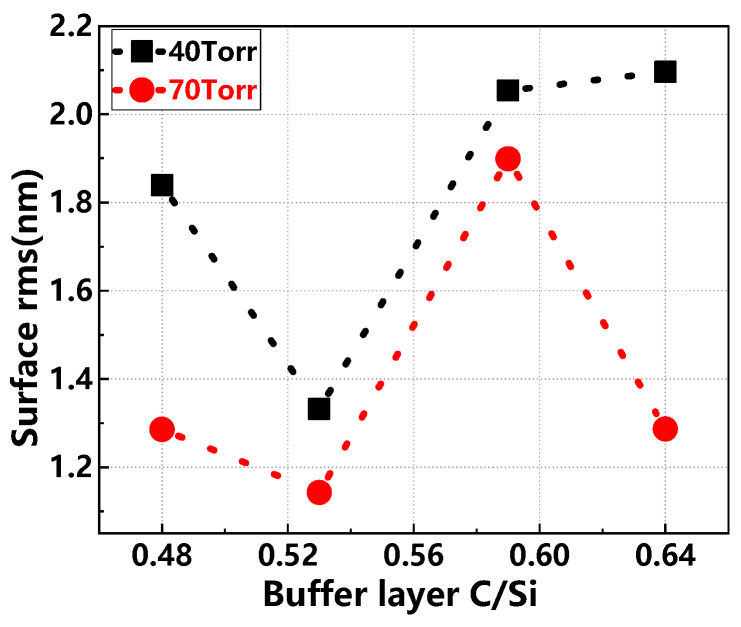
Surface rms values of epitaxial layers for different buffer layer C/Si ratios and growth pressures.

Figure 9 presents the surface rms values of the epitaxial layer under varying buffer layer C/Si ratios and growth pressures. When the buffer layer C/Si ratio is below 0.58, the surface rms follows a similar pattern at both 40 Torr and 70 Torr, initially decreasing and then increasing. As the C/Si ratio increases beyond 0.58, the surface rms remains stable at 40 Torr but continues to decrease at 70 Torr. These observations highlight the substantial impact of the buffer layer C/Si ratio on the epitaxial layer’s surface quality. Adjusting the C/Si ratio can significantly enhance epitaxial wafer uniformity. Furthermore, at 70 Torr, changes in the buffer layer C/Si ratio have a less pronounced effect on the epitaxial layer, facilitating better uniformity, whereas at 40 Torr, its influence is more marked [27,28].

### 3.4. Effect of H_2_ Etching Time on Epitaxial Layer

The quality of the epitaxial layer is influenced by both CVD growth conditions and substrate quality. Untreated substrates often harbor defects like scratches from polishing, which can impair epitaxial growth and device performance. While internal defects are unavoidable, surface damage can be mitigated through in situ hydrogen etching. Studies on 4H-SiC epitaxial growth highlight the importance of surface flatness for crystal replication. Hydrogen etching not only removes scratches but also facilitates step structure formation at optimal temperatures, enhancing epitaxial layer quality. However, at high temperatures, H_2_ etches SiC to produce gaseous hydrocarbons and free silicon. Excessive etching can lead to silicon droplets on the substrate, degrading surface quality. Thus, etching time must be precisely controlled to prevent under- or over-etching and to ensure optimal epitaxial quality.

Based on previous findings, we standardized the buffer layer growth conditions as follows: growth temperature of 1570 °C, pressure of 70 Torr, pre-carbonization time of 3 s, C/Si ratio of 0.53, TCS flow rate of 30 sccm, N_2_ flow rate of 45 sccm, and growth duration of 7 min. The epitaxial layer growth was similarly controlled, with a temperature of 1570 °C, pressure of 70 Torr, C/Si ratio initially set at 0.53 and then adjusted to 0.72 for further studies, TCS flow rate adjusted to 50 sccm, and a growth duration extended to 18 min. We explored the impact of H_2_ etching times of 1, 2, 3, 9, and 12 min on epitaxial quality.

**Figure 10:**
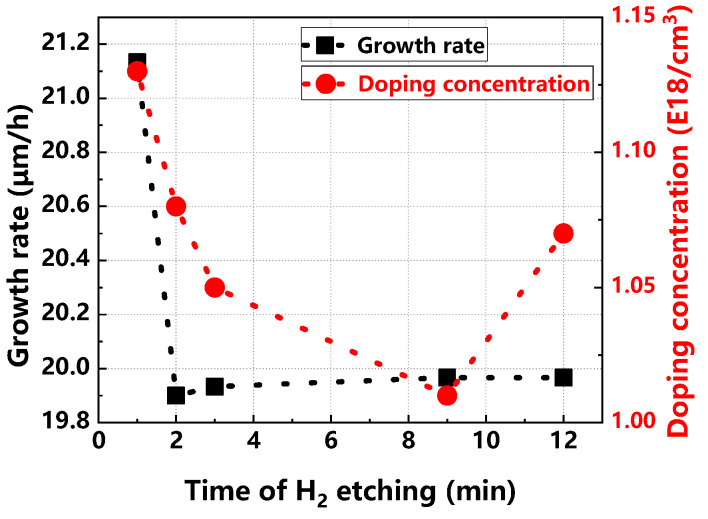
Epitaxial layer growth rate and doping concentration at different H_2_ etching times.

Figure 10 illustrates the effects of varying H_2_ etching times on the epitaxial layer’s growth rate and doping concentration. Initially, as H_2_ etching time increases, the etching rate decreases and then stabilizes. This nonlinear response occurs because, after an etching duration of 2 min, further increases in time do not significantly affect the substrate’s etching depth, leading to a stable growth rate. Regarding doping concentration, it initially declines with increased etching time, then begins to rise. H_2_ etching is critical for removing surface scratches and impurities that can capture dopants or hinder their integration into the lattice, thereby preventing doping inhomogeneity. However, overly prolonged etching can damage the surface, creating areas that negatively impact doping uniformity.

**Figure 11:**
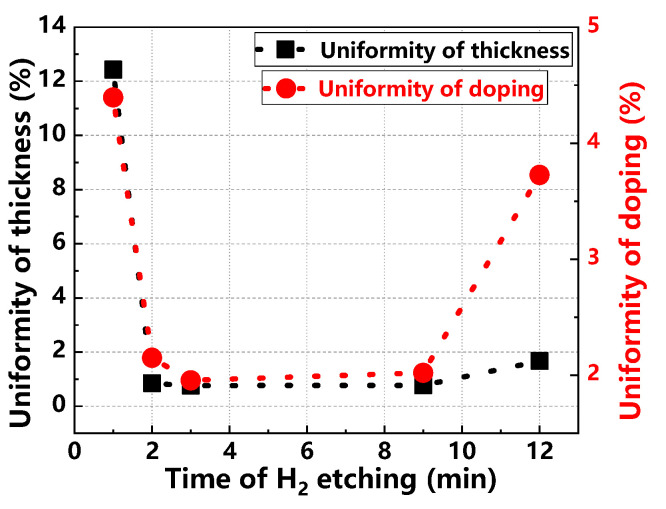
Thickness uniformity and doping uniformity of epitaxial layers at different H_2_ etch times.

**Figure 12:**
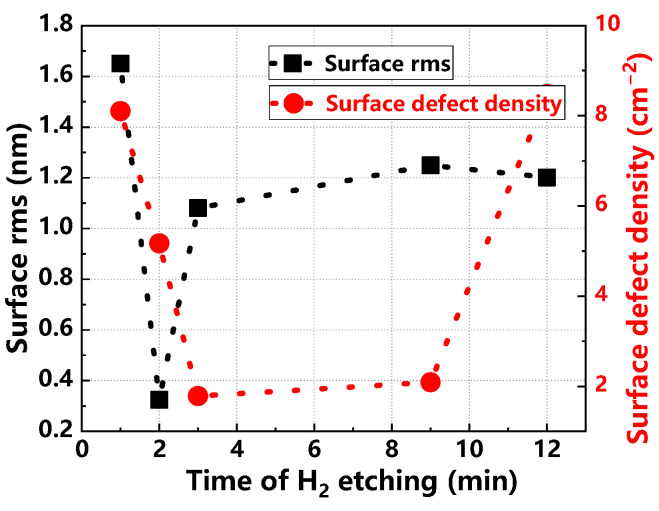
Surface rms values and surface defect density of epitaxial layers at different H_2_ etching times.

Figure 11 details the changes in thickness and doping uniformity of the epitaxial layer at varying H_2_ etching times. Both uniformities first decrease, stabilize, and then improve, achieving optimal levels at a 3 min etching duration. Figure 12 explores H_2_ etching time effects on surface roughness and defect density of the epitaxial layer. Surface roughness follows a pattern of decrease, increase, and then stabilization, with the lowest roughness achieved at 2 min. Similarly, surface defect density decreases and then increases, reaching its lowest at 3 min of etching. These findings indicate that an H_2_ etching time of 3 min yields the highest quality in epitaxial layers [29,30]. Insufficient etching can fail to remove all surface contaminants and damage, leaving residual impurities and roughness that may impede the initial stages of epitaxial growth. This can result in poor nucleation and the introduction of surface defects. Conversely, excessive etching might remove not only the damaged or contaminated layer but also penetrate deeper into the substrate. This over-etching not only wastes material but may also create new surface defects such as pits or excessive roughness, adversely affecting epitaxial growth.

### 3.5. Effect of Growth Pressure on Epitaxial Layer

Experiments were conducted on the growth of epitaxial wafers with buffer layers to examine the impact of growth pressure on the epitaxial layer. The buffer layer’s growth conditions were standardized at a temperature of 1570 °C, a pre-carbonization time of 3 s, an H_2_ etching time of 2 min, a C/Si ratio of 0.53, a TCS flow rate of 30 sccm, N_2_ flow rate of 45 sccm, and a growth duration of 7 min. Subsequently, the buffer and epitaxial layer were grown under varying pressures of 60 Torr, 70 Torr, 75 Torr, and 80 Torr, maintaining a temperature of 1570 °C, a C/Si ratio of 0.72, a TCS flow rate of 50 sccm, and a growth time of 18 min.

**Figure 13:**
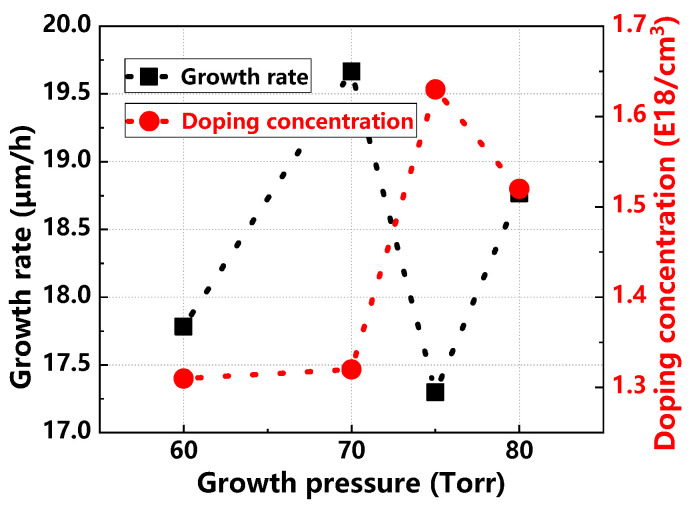
Epitaxial layer growth rate and doping concentration at different growth pressures.

**Figure 14:**
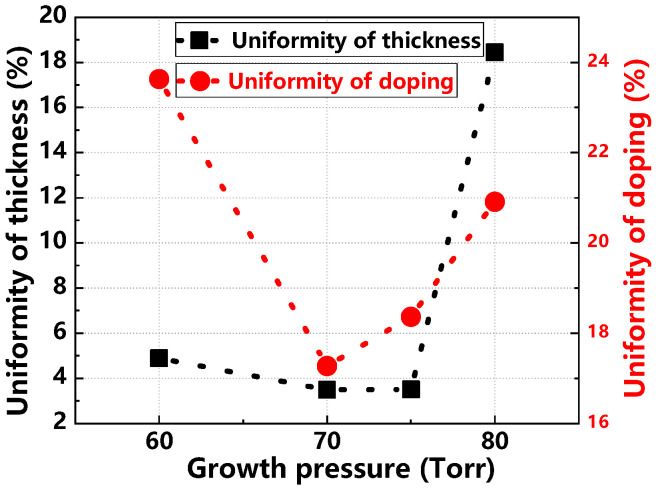
Thickness uniformity and doping uniformity of epitaxial layers at different growth pressures.

Figure 13 and Figure 14 illustrate the variations in growth rate, doping concentration, thickness uniformity, and doping uniformity of the epitaxial layer under different growth pressures. The growth rate fluctuates without a clear pattern as pressure increases. Doping concentration initially increases and then decreases; at lower pressures, it is notably lower due to Si desorption, which increases the actual C/Si ratio on the substrate surface and inhibits nitrogen doping. Thickness uniformity remains stable initially but deteriorates significantly at higher pressures. Similarly, doping uniformity improves initially and then worsens. Optimal thickness and doping uniformity are achieved at a growth pressure of 70 Torr.

**Figure 15:**
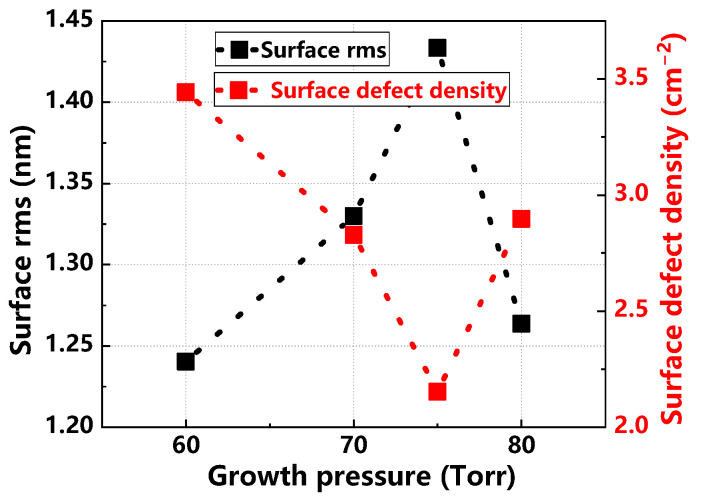
Surface rms values and surface defect density of epitaxial layers at different growth pressures.

Figure 15 illustrates how the surface rms and surface defect density of the epitaxial layer vary under different growth pressures. As growth pressure increases, the surface rms value initially enlarges and then reduces, while surface defect density decreases and subsequently increases. Optimal conditions, with lower surface rms and defect density, are observed at a growth pressure of 70 Torr, where the epitaxial wafers exhibit the highest quality [31,32,33].

## 4. Conclusions

This study systematically investigates the impact of C/Si ratio, pre-carbonization time, H_2_ etching time, and growth pressure on the single buffer and subsequent epitaxial layers during the growth of 6-inch 4H-SiC epitaxial wafers. The findings indicate that a lower C/Si ratio improves thickness uniformity, N_2_ doping efficiency, and doping uniformity in the epitaxial layer. Conversely, a higher C/Si ratio results in a flatter surface and reduced surface rms value in the buffer layer. As for growth pressure, the epitaxial layer’s doping and thickness uniformity first improve and then deteriorate, with optimal uniformity achieved at 70 Torr. At 40 Torr, growth pressure lessens the impact of other parameters on epitaxial quality. Both pre-carbonization and H_2_ etching times significantly influence the uniformity and surface quality of the epitaxial layer, which improve and then decline as these times increase. Optimal results are obtained with a pre-carbonization time of 3 s and an H_2_ etching time of 3 min, effectively stabilizing growth conditions and facilitating the analysis of other growth factors.

## Data Availability

The raw data supporting the conclusions of this article will be made available by the authors on request.
